# Observations on patients with cerebral astrocytoma (glioblastoma multiforme) treated by irradiation under whole-body hypothermia.

**DOI:** 10.1038/bjc.1966.83

**Published:** 1966-12

**Authors:** M. Bloch, H. J. Bloom, J. Penman, L. Walsh


### OBSERVATIONS ON PATIENTS WITH CEREBRAL ASTROCYTOMA (GLIOBLASTOMA MULTIFORME) TREATED BY IRRADIATION UNDER WHOLE-BODY HYPOTHERMIA

M. BLOCH*, H. J. G. BLOOM, J. PENMAN AND L. WALSH

*From the Department of Neurosurgery, Atkinson Morley's Hospital, London, S.W.20, and the Radiotherapy Department, Royal Marsden Hospital, London, S.W.3*

Received for publication July 19, 1966

THE expectation of life for patients with cerebral astrocytomata of high-grade malignancy is very poor with orthodox treatment. Fifty to sixty per cent of patients die within 3 months and 90 per cent within 18 months, whether treatment consists of surgery alone or surgery combined with radiotherapy (Penman, 1961, unpublished data). Preliminary observations describing an apparent increase in radiosensitivity of these tumours, when irradiated under mild whole-body hypothermia, have been reported by us previously (Bloch *et al.*, 1961). It was postulated that reduction in oxygen consumption and increase in dissolved plasma oxygen during hypothermia might lead to increased oxygen availability in the tissues resulting in an improved tumour response.

Our original intention was to conduct a controlled clinical trial to compare the effects of irradiation to the brain at normal and at reduced body temperatures. This was to be carried out in two groups of otherwise similar patients, having proven grades III or IV astrocytomata (classification of Kernohan *et al.*, 1949), for which cerebral decompression, with extensive removal of tumour, had recently been carried out. The next-of-kin of each patient was interviewed, the prognosis for the patient was discussed, and the possible dangers of prolonged hypothermia were clearly explained before permission to treat the patient was accepted.

During hypothermia a marked increase in the response of cerebral tissues to irradiation was observed so that an incident dose of 150 r daily to each side of the skull, covering the entire cerebral hemisphere, was tolerated for only 2 or 3 consecutive days (250 kv ; 15 mA ; H.V.L., 3·4 mm. Cu ; dose rate 32 r per min. ; field size 10 $\times$ 15 cm.). There was also evidence of a differential sensitivity between tumour and normal cerebral tissue, which we thought to favour treatment of the tumour. Owing to the limited dose of irradiation tolerated in cooled patients the controlled clinical trial was abandoned and our efforts were then directed to increasing the total dose of irradiation under hypothermia by giving repeated short courses of treatment at intervals of 7–10 days. The technique of inducing and maintaining hypothermia was described in our previous communication (Bloch *et al.*, 1961).

### OBSERVATIONS

### (a) *Irradiation during a single course of hypothermia*

Irradiated patients developed signs of an acute cerebral reaction which varied from slight drowsiness and confusion, or mild hemiparesis, to deep coma and

* Present address : Institute of Orthopaedics, Stanmore, Middlesex.

flaccid tetraplegia, appearing within 2 to 3 days of commencing irradiation, when the total midline dose to the brain was between 300 to 450 r. These patients were treated with urea (50 g. in 180 ml. water by gastric tube) and the subsequent improvement, usually within one hour, suggested that deterioration had been due to cerebral oedema. In 2 patients even smaller doses of irradiation produced clinical changes: one case received 50 r daily incident to each hemisphere, and the other an initial dose of 50 r increasing by 25 r daily. Both these patients showed evidence of mild generalised depression of the central nervous system, after receiving a total dose of 200 r and 225 r respectively to each side of the skull. Subsequently, some patients were found to tolerate as much as 200 r daily to each side of the skull, but it still was not possible to irradiate them for more than 3 consecutive days without producing a cerebral reaction.

(b) *Irradiation during multiple courses of hypothermia*

During the first cooling episode, 200 r to each hemisphere was given on each of 2 consecutive days. If this was well tolerated, 3 days treatment was given during the second cooling but in some patients this resulted in drowsiness. In this way it was possible to deliver a total incident dose to each hemisphere corresponding to a total midline dose of 3000–4000 r, during a course of 5–7 episodes of hypothermia. The greatest number of times any one patient was cooled was nine. On the few occasions when irradiation was given on 4 instead of 3 consecutive days, gross deterioration occurred leading to stupor or coma and on one occasion contributing to a fatal result. Thereafter irradiation was limited to not more than 3 days during any single episode of hypothermia.

In 4 cases, a control period of hypothermia without irradiation, lasting 4, 6, 6 and 4 days respectively, was undertaken, the intention being to administer the combined treatment of irradiation and hypothermia after an interval of 2 to 3 weeks, if hypothermia alone was well tolerated. In 2 further cases irradiation to the brain was postponed until the 4th and 8th days respectively of continuous hypothermia. None of these 6 patients showed any signs of neurological deterioration and, with one exception, remained well and alert throughout the period of control hypothermia, suggesting that the cerebral reaction to irradiation at a body temperature of 30–33° C. could not be attributed to hypothermia alone. The exception, a woman of 52 years, developed signs of acute circulatory failure on the 4th day of hypothermia and died some 14 hours later. Autopsy revealed evidence of acute pancreatitis with extensive fat necrosis, involving particularly the tail of the pancreas. At first the patient denied the presence of pain or other symptoms and clinical evidence of an intra-abdominal catastrophe could not be found. To avoid masking possible symptoms, pethidine, used to assist in the control of hypothermia, was discontinued. It was only a few hours before death that abdominal pain and muscle guarding appeared.

### RESULTS OF TREATMENT

Thirty patients have been studied. Twenty-eight were treated by irradiation to the brain under hypothermia following surgical decompression; one patient was treated without preliminary decompression, and one (see 2 below) by decompression and hypothermia alone. Ten of these 30 patients died during treatment:

1. The first 3 patients died as a result of the combined effects of irradiation, prolonged hypothermia (18 and 21 days for 2 patients) and pulmonary infection. It was not immediately appreciated that the dose of irradiation, small by conventional standards at normal temperature, could produce such marked effects.

2. One patient received a preliminary period of control hypothermia without irradiation to the brain. This was well tolerated, but whilst waiting to undergo a course of hypothermia with irradiation there were signs of further tumour growth and it was considered unlikely that he would benefit from treatment.

3. Of the remaining 6 patients, 3 died from pulmonary infection, one from multiple causes (irradiation to the brain under hypothermia, acute pancreatitis, secondary infection and rewarming complications), one from acute phlegmonous gastritis and one from acute pancreatitis.

Patient mortality during treatment was high (33%). Even if the first 4 cases are excluded, the mortality is still 23% (6 of 26 cases). The mortality for the total episodes of hypothermia was 10 out of 80 (12·5%) or, excluding the first 4 cases, 6 out of 76 (7·9%).

Twenty patients survived treatment and the details of these are given in Table I. Case 6 was given three courses of treatment, separated by intervals of 7 and 3 months. In case 21 and case 27, treatment under hypothermia was abandoned after a total incident dose of 300 r and 1400 r respectively; in the first this was due to technical difficulties unrelated to the patient, and in the second to acute epididymo-orchitis which took several weeks to resolve. Both

TABLE I.—*Dose and Survival in Thirty Patients Treated by Irradiation under Hypothermia*

| Patient serial number | Sex and age | Episodes of hypothermia | Total days of irradiation | Total dose incident to each side of skull | Died during treatment | Survival after treatment (mths.) | Patient serial number | Sex and age | Episodes of hypothermia | Total days of irradiation | Total dose incident to each side of skull | Died during treatment | Survival after treatment (mths.) |
|---|---|---|---|---|---|---|---|---|---|---|---|---|---|
| 1 | M 37 | 1 | 6 | 850 r | Died | — | *16 | M 41 | 1 | 5 | 750 r | — | 1 |
| 2 | M 42 | 1 | 8 | 1450 r | Died | — | *17 | M 42 | — | — | — | Died | — |
| 3 | F 42 | 1 | 4 | 400 r | Died | — | 18 | M 57 | 5 | 11 | 1650 r | — | 8 |
| 4 | M 49 | 1 | 4 | 350 r | — | 7 | 19 | F 50 | 5 | 11 | 1650 r | — | 5 |
| 5 | F 47 | 1 | 5 | 250 r | — | 4 | 20 | F 49 | 5 | 14 | 2100 r | Died | — |
| 6 | F 46 | 1 | 2 | 300 r | — | 12 | 21 | M 56 | 1 | 2 | 300 r | — | 6 |
| | | 1 | 3 | 450 r | — | 5 | | Plus, at Normal Temp. | | | 3700 r | — | |
| | | 4 | 8 | 1200 r | — | 2 | 22 | M 45 | 6 | 12 | 1800 r | — | 4 |
| 7 | F 56 | 1 | 2 | 300 r | — | 3 | 23 | F 45 | 9 | 20 | 3500 r | — | 5 |
| 8 | F 46 | 1 | 2 | 300 r | — | 3 | 24 | M 56 | 3 | 7 | 1050 r | Died | — |
| 9 | M 50 | 2 | 2 | 300 r | — | 2 | 25 | F 25 | 6 | 17 | 3400 r | — | 7 |
| 10 | F 46 | 1 | 2 | 300 r | — | 3 | 26 | F 37 | 7 | 18 | 3500 r | — | 7 |
| *11 | F 52 | — | — | — | Died | — | 27 | M 37 | 3 | 7 | 1400 r | — | Un-traced |
| | | | | | | | | Plus, at | | | 2550 r | — | |
| *12 | F 40 | 1 | 2 | 300 r | — | 1 | | Normal Temp. and to Superior Skull | | | 1500 r | — | Alive at 11 mths. |
| 13 | M 62 | 1 | 3 | 450 r | — | 4 | 28 | M 54 | 1 | 2 | 400 r | Died | — |
| 14 | F 56 | 1 | 3 | 450 r | — | 3 | 29 | F 27 | 2 | 5 | 1000 r | Died | — |
| 15 | M 53 | 1 | 2 | 300 r | — | 1 | 30 | M 31 | 2 | 5 | 1000 r | Died | — |

* Control period of hypothermia before irradiation.

patients subsequently received supplementary irradiation at normal body temperature.

In Penman's (1961, unpublished) series of 188 patients (grade III and IV astrocytoma) treated at normal body temperature by surgery and post-operative radiotherapy, approximately 50% died within 3 months, 62% within 6 months, 80% within one year and 88% within 18 months. Five (25%) of our patients who survived treatment died within 3 months, 13 (65%) within 6 months and 18 (90%) within one year.

We should like to refer to a number of other observations made during this investigation.

(1) *Special senses.* Five patients who received multiple courses of irradiation under hypothermia developed mild transient deafness and one of these complained also of transient loss of sense of smell and taste. In one patient deafness was noticed after a mid-line dose of 1430 r had been given during three courses of hypothermia, over a total period of 16 days. In 2 patients deafness was noticed after doses of 2000 r and 2800 r had been given in 6 and 5 episodes of hypothermia, respectively. It is probable that in all 3 cases the deafness could have been detected at an earlier stage had this been especially looked for.

(2) *Cerebrospinal fluid protein.* Cerebrospinal fluid protein concentration increased in a number of patients during irradiation under hypothermia, and for the following reasons this was considered the result of cerebral irradiation rather than hypothermia :

(i). In a patient cooled for 6 days without irradiation to the brain, C.S.F. protein concentration was 70 mg. per 100 ml. during the first 4 days of hypothermia and 85 mg. per 100 ml. on the last. This rise is not significant.

(ii). In 4 patients the period of hypothermia was extended after irradiation to the brain had been completed. The C.S.F. protein concentration increased during irradiation, reached a maximum value 1 or 2 days after irradiation was completed, and then fell rapidly despite continued hypothermia. In 1 of these patients the protein concentration increased from 160 to 300 mg./100ml. following irradiation but during the next 2 days fell to 275 and 250 mg./100 ml. respectively. In a second patient the protein increased from 110 to 325 mg./100 ml. and fell to 225 and 120 mg./100 ml. respectively during the next 2 days. In the 3rd and 4th patients the rise in protein was only moderate, from 50 to 100 mg./100 ml. and from 50 to 140 mg./100 ml.

Increase in C.S.F. protein concentration was observed in several other cooled patients during or following irradiation to the brain. In these, however, hypothermia had been terminated within 24 hours of completion of irradiation, and it could not be deduced whether the subsequent fall in protein resulted from cessation of hypothermia or of irradiation. It seems likely, however, that the rise and fall in C.S.F. protein during hypothermia was related to irradiation to the brain. Increase in C.S.F. protein could not be correlated with dose of irradiation, or with the severity of the clinical response to treatment.

In some patients a second rise in C.S.F. protein occurred during rewarming, and this was of interest because of other central nervous system manifestations, namely, rise in C.S.F. pressure, drowsiness and paresis appearing at that time (Bloch, 1965). In the patient described above, in whom C.S.F. protein did not change significantly during 6 days of hypothermia, rewarming was associated with a rise in protein level from 85 to 130 mg./100 ml. within 48 hours, and to

180 mg./100 ml. 3 days later. The patient became drowsy during rewarming but had improved at the time the C.S.F. protein had increased to 180 mg./100 ml. In another patient, cooled for 8 days and irradiated on the first 4 of these, C.S.F. protein rose from 30 to 140 mg./100 ml., but fell to 90 mg./100 ml. by the final day of hypothermia. During rewarming it rose again, to 120 mg./100 ml.

In view of the risks attached to lumbar puncture in patients having supratentorial tumours, C.S.F. examination is not usually made during irradiation of cerebral gliomata, and we have been unable to find information concerning changes in C.S.F. protein during irradiation of the brain at normal body temperature. Since in all our patients a large cerebral decompression had been the initial treatment, we considered lumbar puncture to be safe, particularly during hypothermia, and this proved to be the case.

(3) *Peripheral Blood Changes.* During the first 24–48 hours of hypothermia there is a rise in haematocrit and haemoglobin values and in red cell count. This results from loss of fluid from the intravascular to the interstitial and probably to the intracellular compartments. During prolonged hypothermia these values return towards normal as a result of a reduction in the number of circulating red cells. During rewarming the reverse occurs; i.e. increase in plasma volume precedes rise in circulating red cells, resulting in a temporary fall in haematocrit, haemoglobin and red cell count (Bloch, 1965).

The response of the white blood cells to mild prolonged hypothermia varies. Whereas the number of circulating neutrophils may rise, fall or remain relatively unaltered, lymphocytes and monocytes generally show a profound fall, and basophils and eosinophils may disappear completely, within 24–48 hours. These changes persist throughout the period of hypothermia and may take 2–4 days to return to normal following rewarming (Bloch, 1963).

The platelet count may fall to less than 100,000/c.mm. by the end of 7 to 10 days of hypothermia (Bloch *et al.*, 1961).

(4) *Pancreatitis.* Acute fat necrosis of the pancreas occurred twice during 80 episodes of hypothermia. In one patient (hypothermia without irradiation) this was the immediate cause of death and in the other (hypothermia with irradiation) a contributory cause.

Acute pancreatitis during hypothermia has been described by several observers. The reported incidence of this complication varies and, particularly in accidental hypothermia, may be related to the presence of such complicating factors as coma, respiratory infection, exposure, drugs and injury. Sano and Smith (1940) reported a 10 per cent incidence of pancreatitis as a chance finding at autopsy of patients in whom hypothermia alone was used as a treatment for disseminated carcinoma, and who subsequently died, often after an interval of many months. These authors describe lesions that range from gross foci of fat necrosis and haemorrhage to similar lesions demonstrable only microscopically. It is possible that the actual incidence of microscopic changes during hypothermia was greater than 10%. Duguid, Simpson and Stowers (1961) described 23 patients suffering from accidental hypothermia and found post-mortem evidence of pancreatitis in 5 of 13 of these patients: acute haemorrhage necrosis was present in 2, and focal pancreatic and fat necrosis in 3. Fisher, Fedor and Fisher (1957), in their observations on experimental hypothermia in dogs, found evidence of non-haemorrhagic pancreatitis with associated necrosis of peripancreatic adipose tissue in 10% of their animals in which this organ was examined.

(5) *Acute Epididymo-orchitis.* This occurred in one patient following 3 episodes of hypothermia and irradiation. Urine culture was sterile. Culture and guinea-pig inoculation tests for tubercle bacilli were negative. Although testicular lesions have not been described in clinical reports concerning tissue changes due to hypothermia, Delauney and Lebrun (1954) referred to severe necrotic and pyknotic changes in the lymphoid tissue and testicles of rats cooled to 15° C. for 7 hours. Jones (1962), however, found no evidence of testicular damage in rats cooled for periods up to 27 hours at 20–22° C. Thus, although acute epididymo-orchitis in our case may have resulted from hypothermia, it may have been a complication of catheterisation, despite the lack of evidence of urinary infection.

### DISCUSSION

Under conditions of mild hypothermia (30–33° C., rectal temperature) there is an increase in the sensitivity of cerebral tissues to irradiation. There is some evidence that the response of the tumour may be greater than that of normal brain tissue (Bloch *et al.*, 1961). None of our patients tolerated an incident dose of 200 r daily, to each cerebral hemisphere, for longer than 3 consecutive days. This applied not only to patients who were already drowsy, but also to patients who were fully alert and ambulant before irradiation and who may have already received several courses of irradiation under hypothermia at a lower daily dose rate.

Further evidence for an increased tissue response to irradiation under hypothermia was obtained by Bloom and Dawson (1961) who demonstrated an increased mortality in mice exposed to whole-body irradiation at 31–33° C.

The nature of the increased response of tissues to irradiation under mild hypothermia is unknown. Increased tissue oxygen tension resulting from a lowered metabolic rate, or humoral factors arising from tissue breakdown or endocrine changes are possible explanations. These have been considered elsewhere (Bloom and Dawson, 1961).

At the present time these observations concerning the treatment of intracranial gliomas are largely academic. There is no evidence that life is prolonged as a result of irradiation to the brain under conditions of mild hypothermia. The immediate reponse to irradiation is greater than would be expected at normal body temperature, but subsequently, as at normal temperature, the tumour grows again, leading to death.

The use of induced prolonged hypothermia alone does not necessarily carry an excessive mortality. The mortality from hypothermia combined with cerebral irradiation in cases of brain tumour in this study was 33%, although in terms of 80 episodes of hypothermia it was 12·5%. With the experience now gained it is thought that this can be further reduced. Fay (1941, 1959) used prolonged whole-body hypothermia without irradiation for the treatment of cases of non-cerebral malignancy. He reported 19 deaths in 124 patients during 169 episodes of hypothermia (1–8 days at 27–32° C.), a mortality rate of 15 and 11%, respectively. The high mortality reported in accidental hypothermia (50–80%) is probably related to the factors precipitating this condition, such as drug intoxication and cerebral injury.

### SUMMARY AND CONCLUSIONS

(1) Thirty patients suffering from cerebral glioblastoma multiforme (astrocytoma grades III and IV) have been treated by surgery, followed by irradiation to the brain under conditions of mild whole-body hypothermia (30–33° C.).

(2) Under these conditions there is an increase in the sensitivity of cerebral tissues such that patients will tolerate an incident dose of 200 r daily, to each cerebral hemisphere, for not longer than three consecutive days. There is some evidence that the response of the tumour is greater than that of normal brain tissue. However, life was not prolonged by this treatment.

(3) Ten patients died during treatment. The mortality for the total number of episodes of hypothermia was 12·5%. The high case mortality was considered to be due largely to the combined effects of hypothermia plus irradiation and not from the hypothermia alone.

(4) It is suggested that an increase in radio-sensitivity may also be a property of other tissues of the body during mild whole-body cooling.

(5) Changes in haematocrit and haemoglobin values, in red and white cell count and in platelet count during prolonged hypothermia have been described.

(6) Increase in cerebrospinal fluid protein was observed in a small number of patients during cerebral irradiation under hypothermia, and during rewarming following hypothermia, with or without irradiation.

(7) Five patients developed temporary partial deafness and one of these complained also of impairment of sense of taste and smell following irradiation to the brain under hypothermia.

(8) Acute pancreatitis occurred in 2 patients and acute epididymo-orchitis in one patient during 80 episodes of prolonged hypothermia.

We wish to thank Mr. Wylie McKissock, for his encouragement in carrying out this work. We are greatly indebted to our colleagues at the Royal Marsden Hospital for their help with many clinical problems and special investigations, and to the Matron and Nursing Staff for their considerable efforts in caring for the patients. This work has been supported by grants from the British Empire Cancer Campaign for Research to M.B. and J.P.

### REFERENCES

BLOCH, M.—(1963) *Lancet*, ii, 1255.—(1965) ‘Rewarming following prolonged hypothermia in man’. M.D. Thesis. University of London Library.

BLOCH, M., BLOOM, H. J. G., PENMAN, J. AND WALSH, L.—(1961) *Lancet*, ii, 906.

BLOOM, H. J. G. AND DAWSON, K. B.—(1961) *Nature, Lond.*, **192**, 232.

DELAUNAY, A. AND LEBRUN, J.—(1954) *Annls Inst. Pasteur, Paris*, **86**, 520.

DUGUID, H., SIMPSON, R. G. AND STOWERS, J. M.—(1961) *Lancet*, ii, 1213.

FAY, T.—(1941) *Proc. int. Assembly interst. Post-grad. Ass. N. Am.*, p. 292.—(1959) *J. Neurosurg.*, **16**, 239.

FISHER, E. R., FEDOR, E. J. AND FISHER, B.—(1957) *A.M.A. Archs Surg.*, **75**, 817.

JONES, J. H.—(1962) *Br. J. exp. Path.*, **43**, 257.

KERNOHAN, J. W., MABON, R. F., SVIEN, H. J. AND ADSON, A. W.—(1949) *Proc. Staff Meet. Mayo Clin.*, **24**, 71.

SANO, M. E. AND SMITH, L. W.—(1940) *J. Lab. clin. Med.*, **26**, 443.


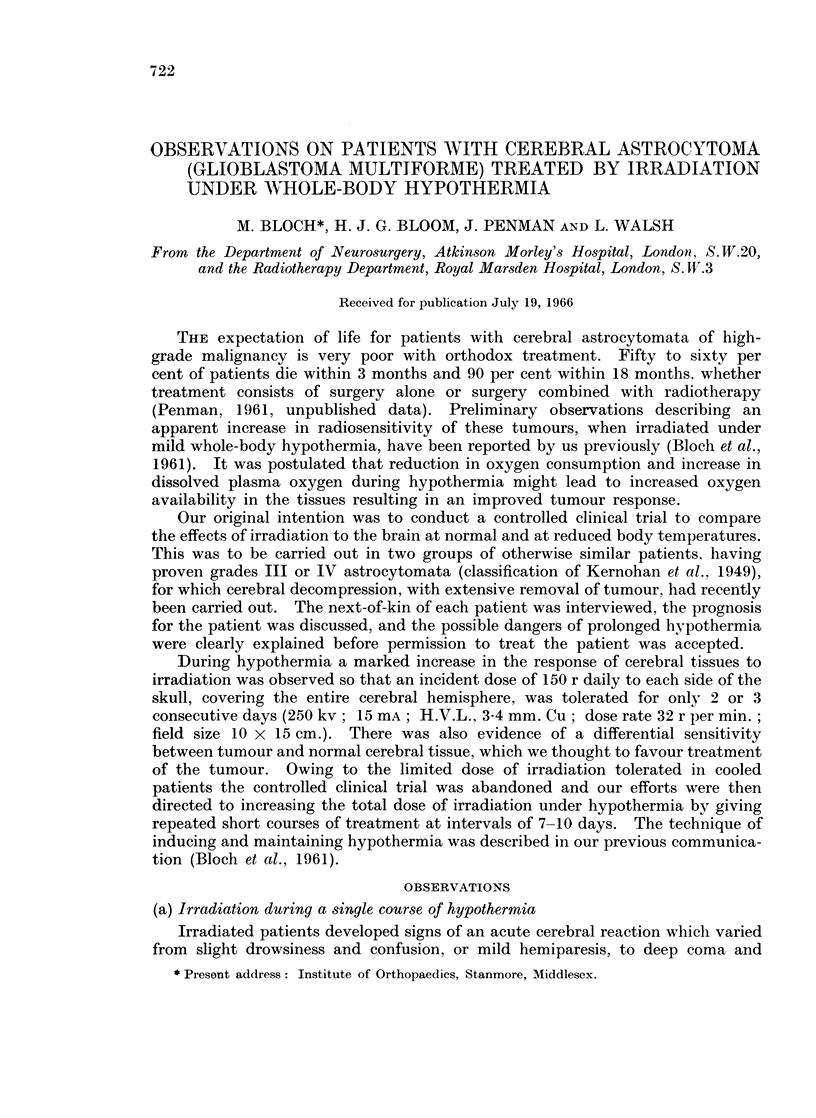

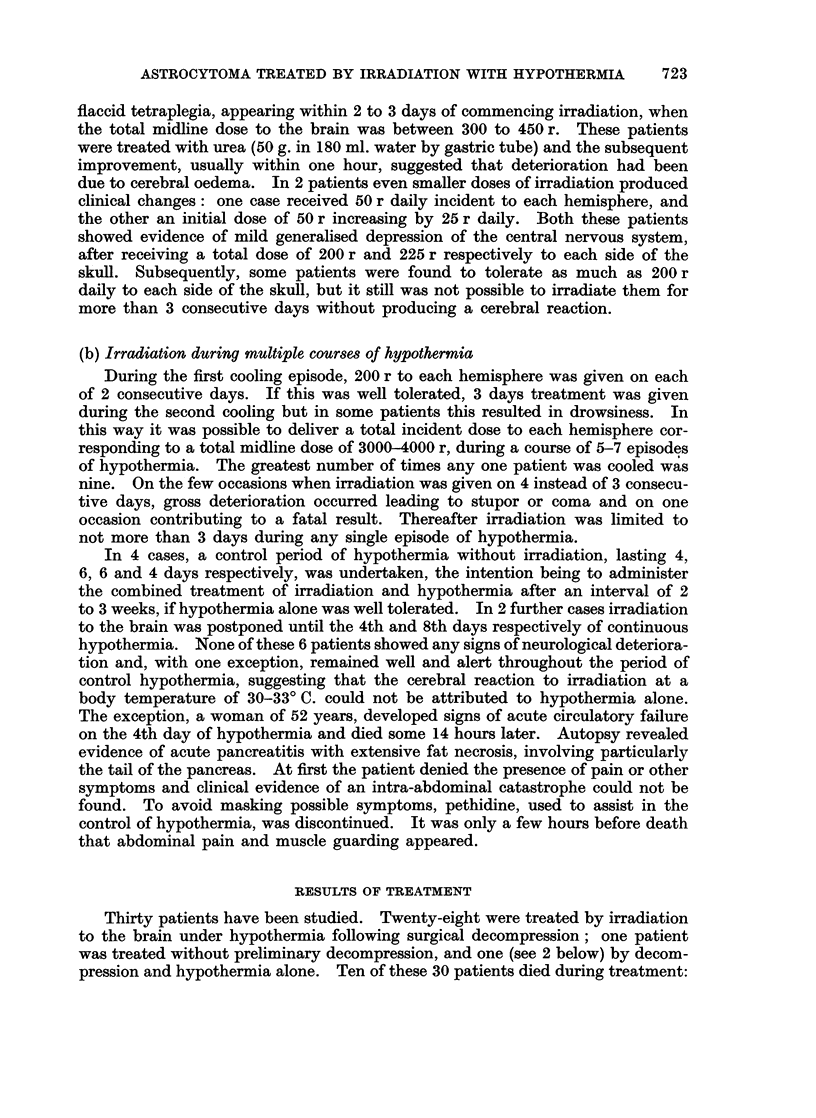

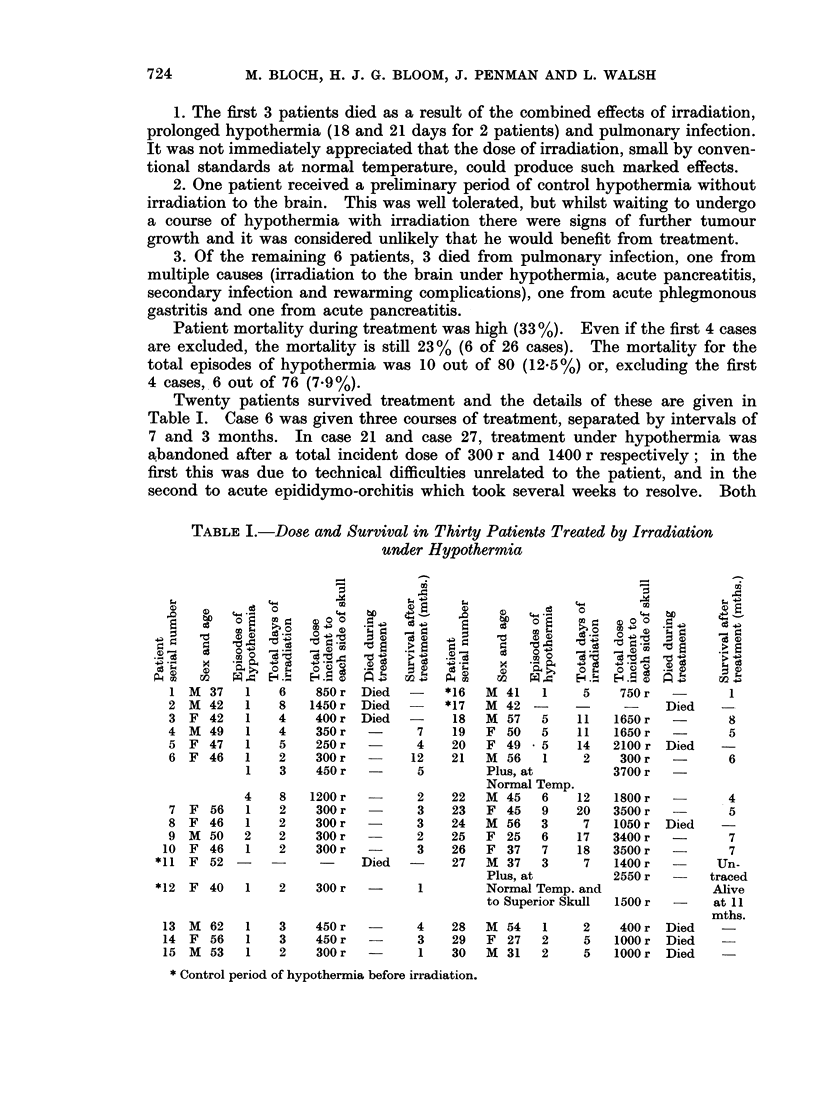

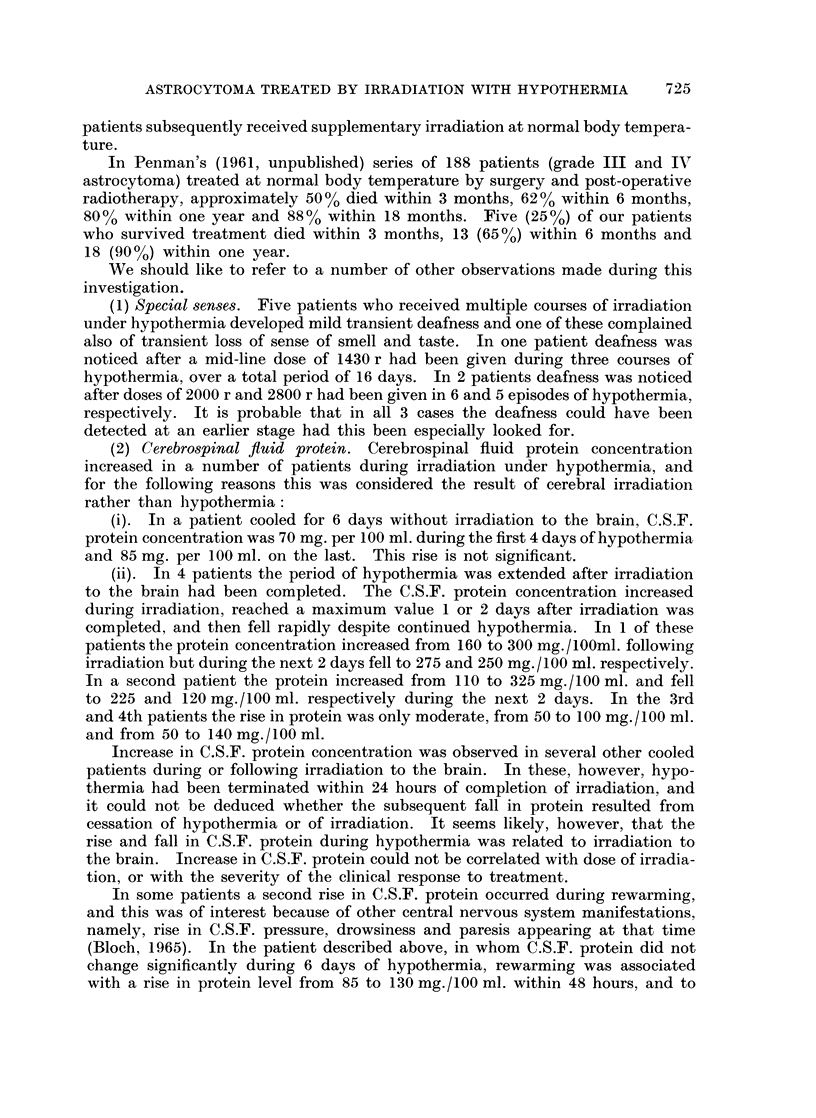

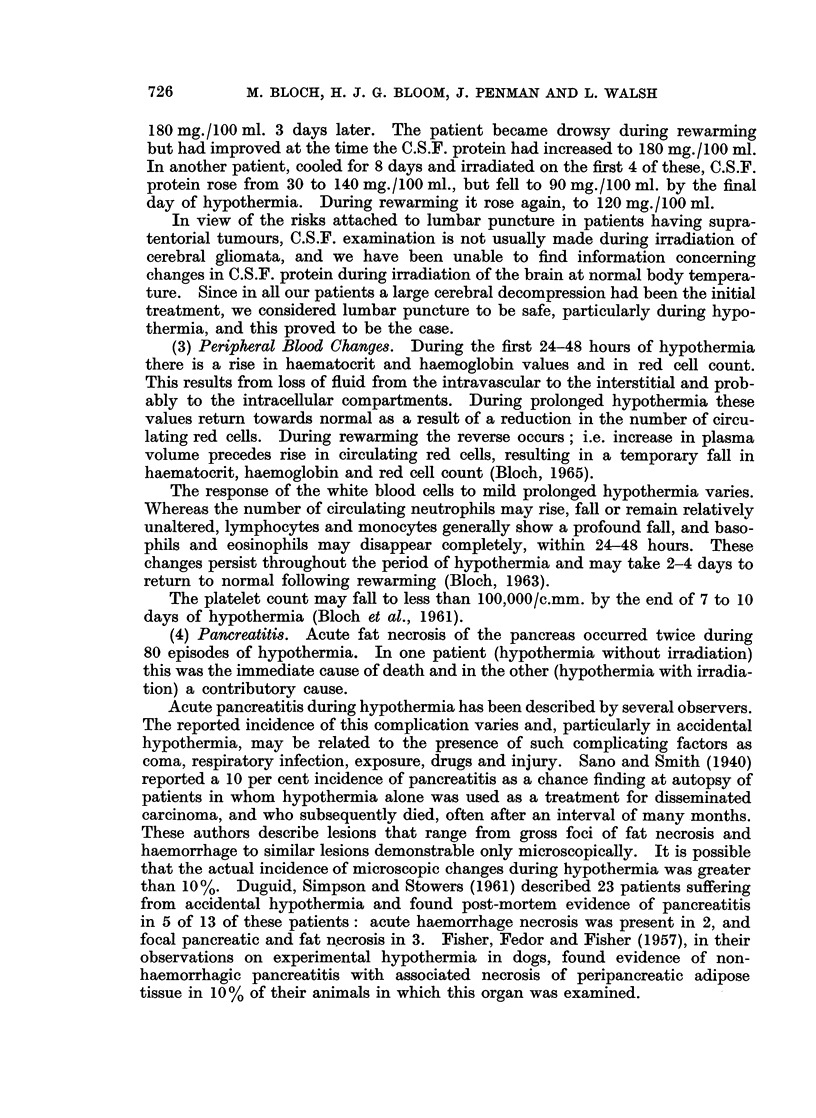

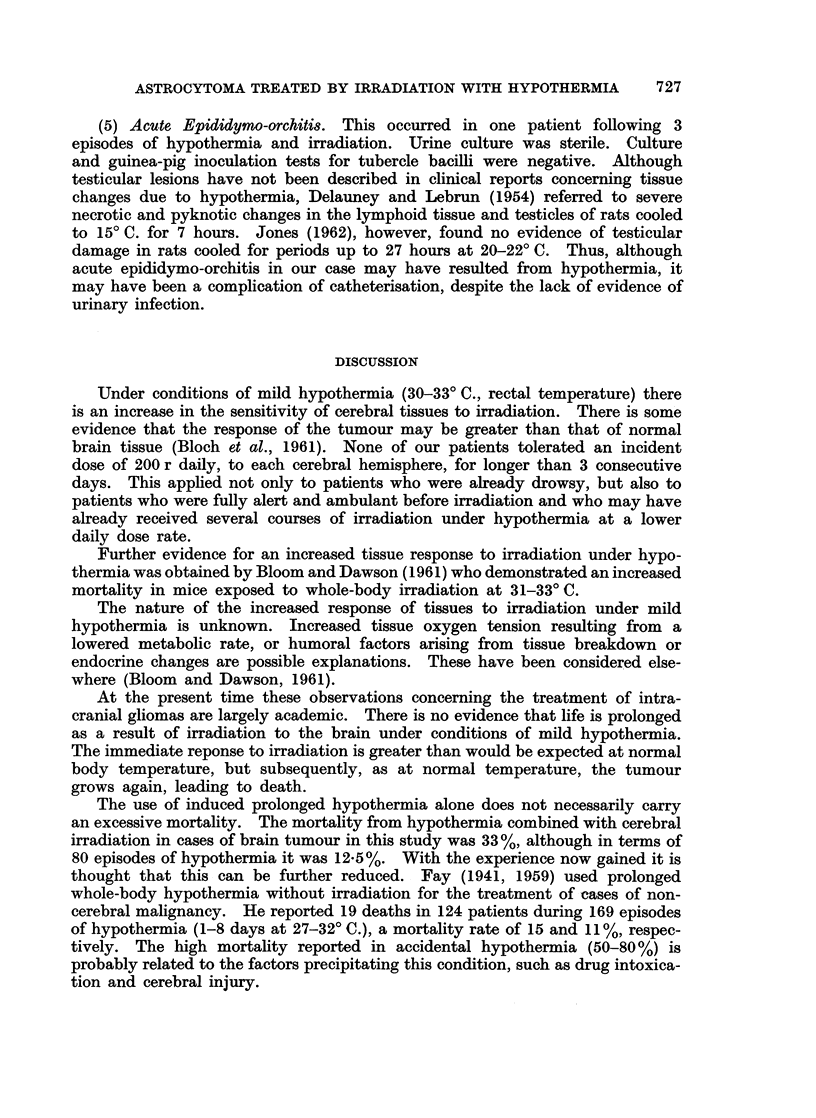

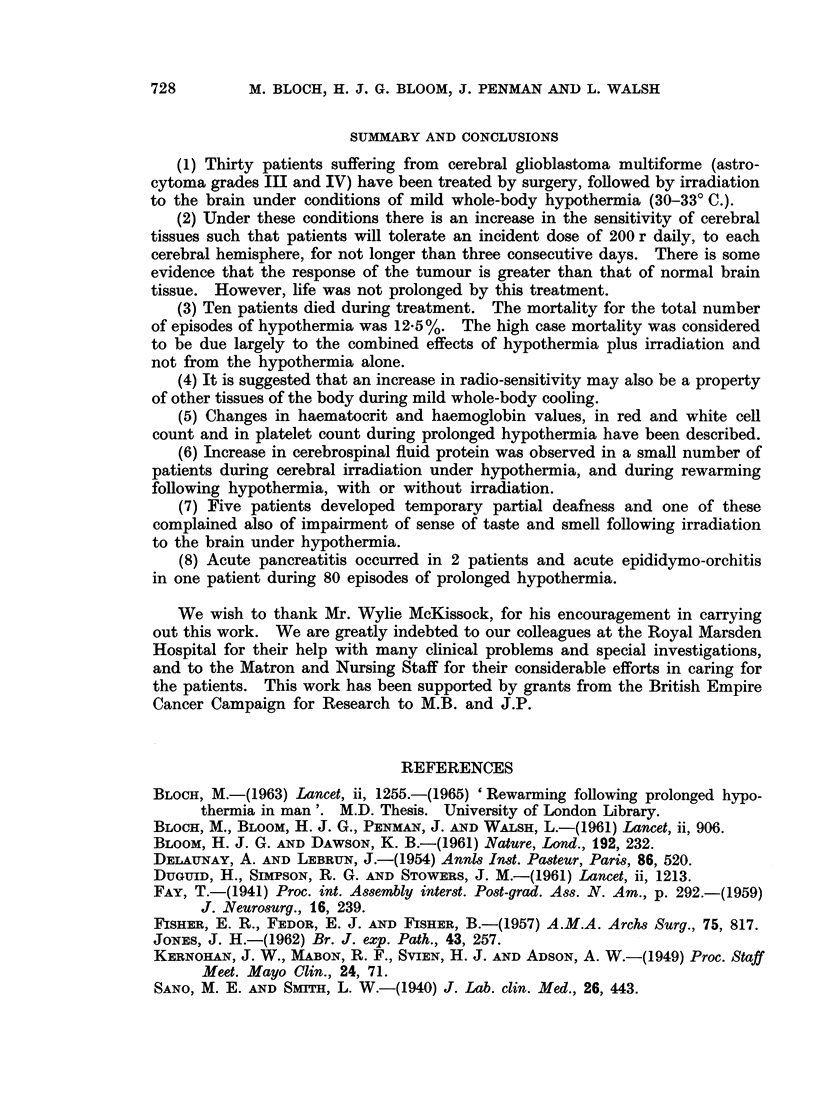


## References

[OCR_00602] BLOOM H. J., DAWSON K. B. (1961). Enhanced effect of total-body x-irradiation in mice under mild hypothermia.. Nature.

[OCR_00604] DELAUNAY A., LEBRUN J. (1954). Inhibition de la diapédèse leucocytaire et lésions viscérales observées chez des animaux soumis a une hypothermie expérimentale.. Ann Inst Pasteur (Paris).

[OCR_00605] DUGUID H., SIMPSON R. G., STOWERS J. M. (1961). Accidental hypothermia.. Lancet.

[OCR_00607] FAY T. (1959). Early experiences with local and generalized refrigeration of the human brain.. J Neurosurg.

[OCR_00611] FISHER E. R., FEDOR E. J., FISHER B. (1957). Pathologic and histochemical observations in experimental hypothermia.. AMA Arch Surg.

